# Predictors of physical activity in older adults 65 years and older: findings in health survey of the Valencian Community

**DOI:** 10.3389/fpubh.2023.1294537

**Published:** 2023-11-27

**Authors:** Silvia Trujillo-Barberá, Pedro García-Martínez, Juana María Sánchez-Martínez, María Ángeles Rodríguez-Herrera, Antonio Ruiz-Hontangas, Javier Gámez-Paya

**Affiliations:** ^1^Department of Health Sciences, Faculty of Health Sciences, Universidad Europea de Valencia, Valencia, Spain; ^2^Nursing School La Fe, Adscript Centre, University of Valencia, Valencia, Spain; ^3^Research Group GREIACC, Health Research Institute La Fe, Valencia, Spain; ^4^Neurology Service, Hospital Doctor Peset, Valencia, Spain; ^5^Biomechanics and Physiotherapy in Sports Research Group (BIOCAPS), Valencia, Spain

**Keywords:** exercise, physical activity, quality of life, mental health, social support, healthy aging, regression analysis

## Abstract

**Background:**

Physical activity is part of a healthy lifestyle in the older adult and is related to multiple variables that promote this behavior.

**Objective:**

To identify the relationship and predictive power of sociodemographic variables, multimorbidity, severity index, risk of poor mental health, social support, affective support and confidential support with the time devoted to physical activity in the population over 65 years of age in the Valencian Community.

**Methods:**

Cross-sectional descriptive analytical study of the data collected in the Health Survey of the Valencian Community on a total of 3,199 people over 65 years of age. The study variables were age, sex, educational level, marital status, social class, multimorbidity, severity index collected with the EQ-5D-5L tool, risk of poor mental health collected with the Goldberg general health questionnaire (GHQ-12), and perceived social, affective and confidential support collected with the Duke-Unc social support scale (Duke-UNC-11).

**Results:**

All variables, except affective support, are significantly related to the time of physical activity performed by people older than 65 years. The severity index has a predictive capacity of 13.7% of physical activity performed and age is able to predict 1.2% of this variable.

**Conclusion:**

Sex, age, education, social class, marital status, multimorbidity, risk of poor mental health or social support and confidentiality are related to the physical activity time of the Valencian population over 65 years of age. On the other hand, the variables severity index and age have been identified as variables capable of predicting up to 14.8% of the variance of the physical activity time variable in our study population.

## 1 Introduction

The aging of the world population is constantly increasing and the population over 60 years of age is expected to double by 2050 (1). This population aging will lead to an increase in age-related chronic diseases. One of the main interventions to prevent and control chronic diseases is the promotion of healthy lifestyles, with physical activity being one of the most relevant (2).

The World Health Organization (WHO) recommends between 150 and 300 min per week of moderate physical activity or between 75 and 150 min per week of intense activity, with the possibility of increasing activity beyond the recommendations to obtain additional benefits. The WHO recommends the same activity for people over 65 years of age as for adults under 65 years of age and advises strength and balance exercises aimed at improving functional capacity and preventing falls. People who achieve these physical activity recommendations are identified as active (3).

Health policy have driven the promotion of physical activity as a desirable lifestyle at all ages and the population has been informed of the benefits of being active. But even so, more than a quarter of the world’s adult population does not achieve a sufficient level of physical activity, with women being less active than men and inactivity affecting higher-income countries to a greater extent (4).

Maintaining an active lifestyle has shown significant benefits among the population over 65 years of age. The reduction of problems related to chronic pathologies such as type 2 diabetes, hypertension, anxiety and depression or insomnia reduces the morbidity of the population and also reduces the risk of falls, cognitive deterioration or overweight, leading to an improvement in the quality of life of the population (3).

The identification of factors associated with inactivity has been extensively studied, but the results have been inconclusive (5). Factors associated with physical inactivity include clinical elements such as chronic pathologies involving pain and fatigue, depression and overweight. At the psychological level, negative perceptions of physical, mental, cognitive or social health, or a low evaluation of the autonomy, independence and self-efficacy of the individual have been related to inactivity. Finally, low socioeconomic level, female sex or low social and family support has also been identified as factors associated with physical inactivity (6–11).

Population health surveys have been carried out in the Valencian Community since 1991, making it possible to obtain valuable information on the level of health and well-being of the population. These surveys have been considered a key instrument for guiding and supporting strategic decisions in the Community’s health policies. The latest Health Survey of the Valencian Community (HSVC), carried out on children and adults, included a sample of the population over 65 years of age, which provides a broad view of this age group in relation to their healthy lifestyles.

The HSVC provides information on many of the variables that may be related to physical activity in the population. Among others, data are collected on age, sex, educational level, marital status, social class, weight, height, the presence of chronic pathologies and other tools such as the quality of life index measured by the EQ-5D-5L, the Goldberg general health questionnaire or the Duke-Unc social support scale. The presence of these variables and the participation of 3,199 people over 65 years of age make this survey a very valuable source of information.

Following WHO recommendations, in the Valencian Community physical activity has been defined as one of the axes of health promotion in the 2022–2030 (12) Health Plan and, within the framework of healthy environments, it has been prioritized as the second most urgent intervention to be implemented, after the promotion of healthy eating habits. In the 2022–2030 health plan, the Valencian Community has set the objective that the active population should reach figures above 33%. For this objective to be achieved in a more efficient way, it is necessary to know how the axes of inequality: sex, age, educational level and social class, and other social factors such as marital status, multimorbidity, perception of mental health or social support influence the practice of physical activity.

For this reason, the aim of this study is twofold: on the one hand, to study the relationship between the variables listed above and the time devoted to physical activity by the Valencian population over 65 years of age and, on the other hand, to identify the variables that are able to predict the time devoted to physical activity in this population.

## 2 Methodology

### 2.1 Participants and procedure

Cross-sectional analytical descriptive study based on data collected in the HSVC 2016. This survey uses a multistage sampling: in the first stage, sampling was performed by family units and in the second stage, all persons in the family unit over 65 years of age, under 14 years of age and 50% of adults between 15 and 64 years of age were selected. To achieve population representativeness, 5,280 family units were necessary, with a standardized distribution by health department and by distribution of rural and urban environment.

The sample selected for this study was 3,199 over 65 years of age who completed the HSVC 2016. The population of people over 65 in the Valencian Community according to the 2016 municipal census was 918,090 people, 18.51% of the total population of the community. To achieve representativeness with a confidence level of 99% and an assumed margin of error of 3%, the sample needed was 1846 people, so the sample was considered to be representative of the total population.

The methodology for data collection in the HSVC 2016 included the participation of a group of surveyors trained for this purpose and coordinated from the Conselleria de Sanitat Universal y Salut Pública (CSUSP) of the Valencian Community. To facilitate the collaboration of the respondents, the CSUSP prepared an explanatory letter that was sent to each household requesting participation in the study. The surveyor was identified at all times by means of a credential at each home visit. The questionnaire was completed by the surveyors, following the responses of the respondents, by means of a registry assisted by a computer application: GANDIA INTEGRA MOBINET. Data collection was carried out between May and December 2016.

### 2.2 Instruments

The questionnaire used for adults had two parts: the first part collected information on the family structure and the second part collected personal information. The questionnaire included 118 questions, divided into 10 sections. In section 1: Perception, the Euroqol-5D instrument in its EQ-5D-5L version (13) was included; in section 2: Morbidity, chronic pathologies diagnosed by a physician were included; in section 3: Mental health, the Golberg General Health Questionnaire (GHQ-12) (14) was included; in section 8: Life habits, we included weight, height and physical activity practice based on the European Health Interview Survey-Physical Activity Questionnaire (EHIS-PAQ) questionnaire (15); in section 9: Living and working conditions, we included the type of work and social support measured using the Duke-UNC tool (16); and in section 10: Sociodemographic characteristics, we included the level of family income.

The age variable was recoded as a qualitative variable, differentiating 3 groups: 65–74 years, 75–84 years, and 85 years or more. The education variable was grouped into four levels: no studies, primary, secondary and university studies. Marital status was divided into 5 groups: single, married, widowed, separated and divorced. Social class was grouped using the classification proposed by the Spanish Society of Epidemiology based on the 2011 National Classification of Occupations (17) and 7 social classes were differentiated, the highest being class I: managers and the lowest class VII: unskilled workers.

The multimorbidity variable was generated from the responses collected in the morbidity section, which included a total of 24 long-term chronic pathologies and an additional free-text option, defined as the clinical situation of a person with two or more chronic pathologies diagnosed by a physician in the last year.

The severity index was calculated using the EQ-5D-5L questionnaire, which presents 5 dimensions (mobility, self-care, activities of daily living, pain or discomfort, and anxiety or depression) and 5 response levels (1 no problem and 5 disabling problem). The severity index is calculated as the sum of the 5 dimensions, minus 5 points and multiplied by 5, with a score range between 0 and 100, with 100 being the highest degree of severity or worst perceived state of health. In the present study, the level of reliability was good, with a Cronbach’s alpha = 0.854 (18).

The quantitative variable risk of poor mental health was calculated using the Goldberg general health questionnaire in its abbreviated 12-question version (GHQ-12). The score range of the questionnaire is between 0 and 12 points. The qualitative variable of risk of poor mental health was recoded into two variables: risk of poor mental health and no risk, considering people with 3 or more points at risk. In the present study, the level of reliability of the questionnaire was good, with a Cronbach’s alpha of 0.889 (18).

The quantitative variables of social support, affective support and confidential support were calculated using the Duke-UNC-11 social support questionnaire, an 11-item questionnaire, with likert-type response from 1 to 5. Perceived social support was evaluated, with 11 being the lowest social support and 55 the highest perceived social support. The questionnaire identified a subscale of confidential support or the possibility of having a person to communicate with, which scored between 7 and 35 points, the higher the score, the more supportive the relationship, and a subscale of affective support or demonstrations of love, affection or empathy in their environment, which scored between 4 and 20 points, with the same interpretation as the previous one (16). The qualitative variables of social, affective and confidential support were recoded as low or normal support using the cut-off points defined for the Duke-UNC-11 questionnaire. The cut-off point for low social support was identified as a score of less than or equal to 32 points, for low affective support as less than or equal to 15 points, and for low confidential support as less than or equal to 18 points (16). In the present study, the level of reliability of this questionnaire was excellent with a Cronbach’s alpha of 0.919 (18).

The quantitative variable “activity time” was obtained from three of the items of the EHIS-PAQ questionnaire and the variable “physical activity time” was calculated as the sum in minutes per week of the items: how much time do you walk to get around, how much time do you usually use a bicycle to get around, and how much time do you spend practicing sports, gymnastics, cycling, walking fast, at least 10 min at a time, in a normal week?

### 2.3 Analysis of data

In the descriptive study, qualitative variables were presented as absolute numbers and percentages and quantitative variables as mean and standard deviation or median and interquartile range according to their normal or non-normal distribution, respectively.

To ensure the internal consistency of the measurements in this study, the level of reliability for the scales used was analyzed using Cronbach’s alpha coefficient for EQ-5D-5L, GHQ-12 and Duke-UNC-11. Reliability was estimated to be excellent with an alpha greater than 0.90, good between 0.80 and 0.89, and acceptable between 0.70 and 0.79 (18).

For the bivariate or correlational analysis, a normality analysis was performed on the distribution of the quantitative variables using the Kolmogorov–Smirnov test, with all variables showing a non-normal distribution. For the correlational study of the quantitative variables in relation to physical activity time, Spearman’s S was used. For the bivariate analysis of dichotomous variables, the Mann–Whitney U test was used for independent samples and the Kruskal-Wallis analysis for polytomous variables. In all tests, a p less than 0.05 was considered as the level of statistical significance.

Finally, a linear regression analysis using the stepwise method was used to identify the variables with predictive capacity for the time of physical activity performed. Those variables that showed significance in the previous analyses were included in the model as independent variables. The principle of parsimony was applied to the models obtained in order to identify as the most appropriate model the one that would explain the proposed explanatory variables in the simplest way and under equal conditions (19). Finally, and following Cohen’s criteria (20), we considered that an R-squared value of less than 0.1 did not show relevant predictive capacity, an R-squared value between 0.1 and 0.25 showed dependence in the explanation of the variance of the variable, and with an R-squared value greater than 0.25 we could affirm that the predictive model was clinically relevant.

All analyses were performed with the SPSS Statistics for Windows statistical package (IBMSPSS Statistics for Windows, Version 23.0. IBM Corp, Armonk, NY, USA).

### 2.4 Ethical considerations

The present investigation complies with the ethical precepts formulated in the Declaration of Helsinki of the World Medical Association on ethical principles for medical research involving human subjects and its subsequent revisions, as well as those required by the applicable regulations according to the characteristics of the study, and was approved by the Research Ethics Committee of the General Directorate of Public Health and the Higher Center for Public Health Research of the Valencian Community on January 9, 2020, with opinion number 20200109/06.

## 3 Results

Of the 3,199 HSVC 2016 participants over the age of 65 years, 62.7% (*n* = 2007) were women, with a mean age of 80.04 years. The mean age of men was 79.71 years. By age, 18.7% (*n* = 598) belong to the 65–74 year old group, 56.7% (*n* = 1813) to the 75–84 year old group and 24.6% (*n* = 788) to the 85 or older group. The sample had no primary education 47.8% (*n* = 1,529), 52% (*n* = 1,665) were married, 31.7 and 15.5% (*n* = 1,013 and 496) belonged to social class V and VI (skilled in the primary sector, semi-skilled and unskilled), 71.7 and 15.5%, respectively, (*n* = 1,013 and 496), 71. 7% had multimorbidity (*n* = 2,293), 38.4% had poor mental health risk (*n* = 1,227), 4.4% had low social support (*n* = 141), 8.3% had low affective support (*n* = 266) and 7.3% had low confidential support (*n* = 235).

Table 1 shows the analysis of the average time of physical activity performed by the participants in the study grouped by qualitative variables. The people who were most physically active were men, in the 65–74 age group, with higher educational levels, separated or divorced, without multimorbidity and with normal social and confidential support. In all cases, there were significant differences.

**Table 1:** Median, interquartile range and results of Mann–Whitney U and Kruskall-Wallis H tests for qualitative variables related to physical activity time.

		Median^*^	IQR (p25–p75)^*^	*p*
Gender	Man	220	465	<0.001^U^
	Woman	120	315	
Age groups	65–74	294.5	407.05	<0.001^H^
	75–84	180	390	
	≥85	37.5	210	
Studies	No education	95	322.5	<0.001^H^
	Primary	210	370	
	Secondary	275	405	
	University	240	360	
Marital status	Single	140	390	<0.001^H^
	Married	210	380	
	Widowed	120	300	
	Separated	247.5	678.75	
	Divorced	350	702.5	
Social class	I	210	467.5	<0.001^A^
	II	210	350	
	III	210	388.75	
	IV	180	390	
	V	180	410	
	VI	180	370	
Multimorbidity	No	280	397.5	<0.001^U^
	Yes	140	420	
Poor mental health risk	Yes	105	360	<0.001^U^
	No	210	350	
Social support	Low	120	285	0.043^U^
	Normal	180	390	
Emotional support	Low	150	376.25	0.452^U^
	Normal	180	400	
Confidential support	Low	120	295	0.048^U^
	Normal	180	390	

^*^Median and IQR are presented as minutes per week; ^H^: Kruskal-Wallis H; p: significance level of the statistical test; IQR: interquartile range; ^U^: Mann–Whitney U test.

Table 2 shows the description and analysis of the quantitative variables and the Spearman correlation coefficient in relation to physical activity time. It stands out that age, severity index and risk of poor mental health were significantly related to physical activity (*p* < 0.001). All variables were inversely related to the time spent in physical activity, with less time spent in physical activity the higher the score of the variables studied.

**Table 2:** Median, interquartile range and Spearman correlation of physical activity time with quantitative variables.

	*N*	Median	IQR	S	*p*
Time of activity (minutes per week)	3,030	210	390		
Age	3,199	80	8	−0.331	<0.001
Severity index	3,163	15	25	−0.536	<0.001
Poor mental health risk	3,199	1	4	−0.251	<0.001
Social support	2,708	48	11	−0.006	0.774
Affective support	2,776	21	6	−0.019	0.321
Confidential support	2,936	27	6	−0.019	0.314

p: Spearman’s S correlation test significance level; RIC: interquartile range; S: Spearman’s S correlation test result.

Table 3 shows the linear regression model in which the variables, both qualitative and quantitative, that had shown statistical significance in the nonparametric mean comparison and correlation analyses (Tables 1, 2) were included. This model returned a predictive capacity of 14.8% of the physical activity performed by the population over 65 years of age using only two variables: severity index and age. Following the principle of parsimony, this was the simplest predictive model applicable to our population.

**Table 3:** Linear regression model for physical activity time.

	Beta	*p*	R^2^	F
Model information		<0.001	14.8%	202.588
Severity index	−0.335	<0.001	13.7%	
Age	−0.115	<0.001	1.2%	

Beta: predictive power of the independent variable F: model adjust P: significance level R^2^: Percent Variance Explained Variables excluded from the model: gender, age groups, studies, marital Status, social class, multimobidity, poor mental health, social support, confidential support and poor mental health risk.

## 4 Discussion

Our sample has a mean age (79.91 years) higher than most of the studies reviewed on physical activity in the population over 65 years of age. The mean age of these studies was less than 70 years (21–23) or less than 80 years (24, 25). The high representation of the female population in our sample (62%) was similar to that of other studies, which presented samples with more than 60% of women studied (21–25).

The axes of gender, age and social class inequality have been related to a lifestyle in which less physical activity is included in daily routines. In the European context, contradictory data have been presented in the older adult, showing that in Germany women over 50 years of age do less physical activity than men (26) and in the Spanish context, women over 65 years of age do more physical activity than men (27). This difference could be explained by the hypothesis that younger women have a greater workload related to the upbringing and education of their children and that as they get older the differences between sexes could diminish or, as in the case of the Spanish study, be reversed (27). On the other hand, aging presents a decline in the attributes of strength, resistance or endurance that has been associated with less physical activity in older age groups. These hypotheses are supported by the results of a meta-analysis showing a higher probability of being inactive in the female group (OR = 1.47<; 95% CI: 1.15–1.89) or among the older population (OR = 1.27; 95% CI: 1.13–1.42) (28). All these data are consistent with the results of our study showing lower physical activity among women and in older age groups (over 75 years).

On the other hand, social class has also been related to lower physical activity, with a higher probability of being inactive in the population with low economic income (OR = 1.12; 95% CI = 1.02–1.43) (28, 29). Following the National Classification of Occupations 2011 (17) social classes IV, V, and VI identify social classes with lower purchasing power, they presented less physical activity time than high social classes. The intersectoral relationship of the social determinants of health and the axes of inequality show an interdependent relationship and explain how the social position generated by macro-social structures is reflected in the micro-social and individual context (30).

Multimorbidity has been shown to be a variable that is inversely related to physical exercise. A meta-analysis studying the relationship between physical activity and multimorbidity showed that the probability of physical exercise in the older adult population with multimorbidity is OR = 0.81 (95% CI = 0.73–0.89) (31). This relationship has been controversial in the literature, since some authors identify morbidity associated with pathologies that cause pain or fatigue as a factor associated with lower adherence to physical activity (7) and other authors refute this idea by not identifying differences between people with cardiovascular disease or diabetes, which are not usually associated with pain or fatigue (32). This controversy would require specific studies in order to be resolved. In our study, the data are in line with the results presented in the aforementioned meta-analysis (31), but no differentiation has been made between types of pathologies that cause pain or fatigue, so that our results could not enter into discussion with the aforementioned controversy.

Self-perception of health is another factor that has been associated with physical activity in the population. Thus, a meta-analysis shows that there is a greater probability of inactive behavior in the population with the worst perception of health (OR = 1.48; 95% CI = 1.09–2.02) (28) and in the Spanish context, the probability of being inactive in the population with the worst perception of their own health was OR = 2.45, 95% CI = 1.85–3.24, in a study carried out in Las Palmas de Gran Canaria (33). Self-perception of health assesses a concept similar to the severity index, so in our study it is the severity index measured with the EQ-5D-5L questionnaire, which shows a significant correlation that relates a worse perception of health with less physical activity, as indicated in previous studies. These data coincide with the results of other studies, identifying the higher severity index as one of the main factors negatively related to the performance of physical activity (5, 34, 35). Although the accuracy of the measurement of quality of life in the older adult using the EQ-5D-5L questionnaire has been questioned, suggesting its extension to other aspects of quality of life such as social contact, perception of the treatment received by others and level of independence, this tool has been recognized as adequate for comparing health-related quality of life among the older adult (36).

The population at risk of mental health problems or with a low perception of social support have been identified as populations with a hands-on lifestyle (37, 38). These authors hypothesize that the greater perception of social support is related to the person’s optimism and self-esteem, these three elements being protective factors against the risk of presenting mental health problems. The data from our study show differences in the time dedicated to physical activity compared to the risk of poor health, with physical activity being lower both in the group classified as a population at risk of poor mental health and with the quantitative variable risk of poor health measured with the GHQ-12. The significant differences found in our population associating low social support and low confidential support with less time spent in physical activity are in line with the proposed hypothesis of a link between the two variables, as proposed by the authors (37, 38).

Finally, the regression model has identified the variables: severity index and age as those with the greatest predictive capacity related to physical activity. These two variables predict up to 14.8% of the variance of the time spent in physical activity by the older adult in the Valencian Community and show that there is a dependence in the explanation of the variables, following the guidelines established by Cohen (20).

One of the limitations of this study is the possibility of extrapolating these results to contexts other than the Valencian Community. The effects that social and economic conditions exert on the health of the population and the distribution of these determinants generate inequalities in health and in the possibility of adopting healthy lifestyles (39), so that in other populations with different social and economic conditions the results could be different. On the other hand, the handling of the concepts of sedentary lifestyle and physical inactivity presents some difficulties in scientific studies related to nursing, since this discipline recognizes physical inactivity as a diagnosis, catalogued by the NANDA International (NANDA-I) classification as Sedentary Lifestyle (SL) and defined as a lifestyle characterized by a low level of physical activity. Thus, some publications may use sedentary lifestyle, low level of physical activity and insufficient physical activity as synonyms in their publications (28).

The results obtained allow us to propose new studies to evaluate the effectiveness of the interventions proposed in the Valencian Community to reduce health inequalities. These interventions incorporate the health perspective in the planning of public policies in non-health sectors, such as housing, labor, education or environmental policies, among others. Among the recommendations proposed for these interventions, grants were included for local entities and non-profit organizations in the Valencian Community that promote the integration of groups with diversity through physical activity and sport (39). Finally, as the severity index has been identified as the main predictor of the time spent in physical activity by the population of the Valencian Community, if a population-based intervention based on the individual is desired, it would be necessary to take it into account. Thus, as the severity index is an individual perception of one’s own health, before making any recommendation or prescription of physical activity to the population over 65 years of age, it would be necessary to carry out a prior assessment of the barriers and facilitators for the development of health-related physical activity and the willingness of the individual to change his or her behavior. These variables have been identified as a strong predictor of adherence to physical exercise in people with chronic pathologies (40). Barriers and facilitators should be openly discussed at the initial assessment of the intervention and at subsequent intervals to improve the individualization of exercise prescription (41). Follow the self-determination model (11) and promote self-determined forms of motivation that have shown greater adherence to physical exercise (42).

From all that has been described so far, we can conclude that the variables: sex, age, education, social class, marital status, multimorbidity, risk of poor mental health or social support and confidentiality are related to the time dedicated to physical activity in the Valencian population over 65 years of age, and on the other hand, the variables: severity index and age have been identified as variables capable of predicting up to 14.8% of the variance of the physical activity time variable in our study population.

## Data availability statement

The data analyzed in this study is subject to the following licenses/restrictions: the data are property of the health department of the valencian community and this institution is responsible for the access to them. Requests to access these datasets should be directed to epidemiología_dgsp@gva.es.

## Ethics statement

The studies involving humans were approved by Research Ethics Committee of the General Directorate of Public Health and the Higher Center for Public Health Research of the Valencian Community on January 9, 2020, with the opinion number 20200109/06. The studies were conducted in accordance with the local legislation and institutional requirements. Written informed consent for participation was not required from the participants or the participants' legal guardians/next of kin in accordance with the national legislation and institutional requirements. Written informed consent was obtained from the individual(s) for the publication of any potentially identifiable images or data included in this article.

## Author contributions

ST-B: Conceptualization, Formal Analysis, Investigation, Methodology, Writing – original draft, Writing – review & editing. PG-M: Data curation, Methodology, Software, Writing – original draft, Resources. JS-M: Data curation, Resources, Writing – original draft. MR-H: Methodology, Software, Writing – review & editing. AR-H: Funding acquisition, Writing – review & editing. JG-P: Conceptualization, Formal Analysis, Investigation, Writing – review & editing.

## References

[1] World Health Organization (2015). Informe mundial sobre el envejecimiento y la salud. Available at: https://apps.who.int/iris/bitstream/handle/10665/186466/9789240694873_spa.pdf [Accessed December 9, 2022].

[2] Mora R. Medicina del estilo de vida: la importancia de considerar todas las causas de la enfermedad. Rev Psiquiatr Salud Ment. (2012) 5:48–52. doi: 10.1016/j.rpsm.2011.04.002 22854504

[3] Bull FC al-Ansari SS Biddle S Borodulin K Buman MP Cardon G. World Health Organization 2020 guidelines on physical activity and sedentary behaviour. BJSM. (2020) 54:1451–62. doi: 10.1136/bjsports-2020-102955, PMID: 33239350 PMC7719906

[4] World Health Organization (2023). Actividad Física. Available at: https://www.who.int/es/news-room/fact-sheets/detail/physical-activity#:~:text=La%20inactividad%20f%C3%ADsica%20es%20uno,nivel%20suficiente%20de%20actividad%20f%C3%ADsica [Accessed April 18, 2023].

[5] Collado-Mateo D Lavín-Pérez AM Peñacoba C del Coso J Leyton-Román M Luque-Casado A. Key factors associated with adherence to physical exercise in patients with chronic diseases and older adults: an umbrella review. IJERPH. (2021) 18:2023. doi: 10.3390/ijerph18042023, PMID: 33669679 PMC7922504

[6] Bachmann C Oesch P Bachmann S. Recommendations for improving adherence to home-based exercise: a systematic review. Phys Med Rehabil Kurortmed. (2017) 28:20–31. doi: 10.1055/s-0043-120527

[7] Catala P Lopez-Roig S Ecija C Suso-Ribera C Peñacoba Puente C. Why do some people with severe chronic pain adhere to walking prescriptions whilst others won’t? A cross-sectional study exploring clinical and psychosocial predictors in women with fibromyalgia. Rheumatol Int. (2021) 41:1479–84. doi: 10.1007/s00296-020-04719-w, PMID: 33048198

[8] Leone LA Ward DS. A mixed methods comparison of perceived benefits and barriers to exercise between obese and non-obese women. J Phys Act Health. (2013) 10:461–9. doi: 10.1123/jpah.10.4.461, PMID: 23714626 PMC3904548

[9] Vseteckova J Deepak-Gopinath M Borgstrom E Holland C Draper J Pappas Y. Barriers and facilitators to adherence to group exercise in institutionalized older people living with dementia: a systematic review. Eur Rev Aging Phys Act. (2018) 15:11–1. doi: 10.1186/s11556-018-0200-3, PMID: 30455778 PMC6225693

[10] Room J Hannink E Dawes H Barker K. What interventions are used to improve exercise adherence in older people and what behavioural techniques are they based on? A systematic review. BMJ Open. (2017) 7:e019221. doi: 10.1136/bmjopen-2017-019221, PMID: 29247111 PMC5736048

[11] Ryan RM Deci EL. Self-determination theory and the facilitation of intrinsic motivation, social development, and well-being. Am Psychol. (2000) 55:68–78. doi: 10.1037//0003-066x.55.1.68, PMID: 11392867

[12] Dirección General de Salud Pública y Adicciones (2022). Servicio de Planificación y evaluación de políticas de Salud. V Plan de Salud 2022–2030. Generalitat Valenciana. Conselleria de Sanitat Universal i Salut Pública.

[13] Herdman M Gudex C Lloyd A Janssen MF Kind P Parkin D. Development and preliminary testing of the new five-level version of EQ-5D (EQ-5D-5L). Qual Life Res. (2011) 20:1727–36. doi: 10.1007/s11136-011-9903-x 21479777 PMC3220807

[14] Sánchez-López MP Dresch V. The 12-item General health questionnaire (GHQ-12): reliability, external validity and factor structure in the Spanish population. Psicothema. (2008) 20:839–43. PMID: 18940092

[15] Finger JD Tafforeau J Gisle L Oja L Ziese T Thelen J. Development of the European health interview survey - physical activity questionnaire (EHIS-PAQ) to monitor physical activity in the European Union. Arch Public Health. (2015) 73:59–11. doi: 10.1186/s13690-015-0110-z, PMID: 26634120 PMC4667448

[16] Bellón Saameño JA Delgado Sánchez A Luna del Castillo J Lardelli Claret P. Validez y fiabilidad del cuestionario de apoyo social funcional Duke-UNC-11. Aten Primaria. (1996) 18:153–63. PMID: 8962994

[17] Domingo-Salvany A Bacigalupe A Carrasco JM Espelt A Ferrando J Borrell C. Propuestas de Clase Social neoweberiana y neomarxista a partir de la Clasificación Nacional de Ocupaciones 2011. Gac Sanit. (2013) 27:263–72. doi: 10.1016/j.gaceta.2012.12.009, PMID: 23394892

[18] Hunsley J Mash EJ. A guide to assessments that work. New York: Oxford University press (2008).

[19] Hair JF Black WC Babin BJ. Multivariate data analysis: A global perspective. Boston: Pearson (2010).

[20] Cohen J. Statistical power analysis for the behavioral sciences. Hillsdale, NJ: L. Erlbaum Associates (1988).

[21] French DP Olander EK Chisholm A Mc Sharry J. Which behaviour change techniques are most effective at increasing older adults' self-efficacy and physical activity behaviour? A systematic review. Ann Behav Med. (2014) 48:225–34. doi: 10.1007/s12160-014-9593-z, PMID: 24648017

[22] Hobbs N Godfrey A Lara J Errington L Meyer TD Rochester L. Are behavioral interventions effective in increasing physical activity at 12 to 36 months in adults aged 55 to 70 years? A systematic review and meta-analysis. BMC Med. (2013) 11:1–12. doi: 10.1186/1741-7015-11-75, PMID: 23506544 PMC3681560

[23] O’Brien N McDonald S Araújo-Soares V Lara J Errington L Godfrey A. The features of interventions associated with long-term effectiveness of physical activity interventions in adults aged 55±70 years: a systematic review and meta-analysis. Health Psychol Rev. (2015) 9:417–33. doi: 10.1080/17437199.2015.1012177, PMID: 25689096

[24] Chase JA. Interventions to increase physical activity among older adults: a meta-analysis. Gerontologist. (2015) 55:706–18. doi: 10.1093/geront/gnu090 25298530 PMC4542588

[25] van der Deijl M Etman A Kamphuis CB van Lenthe FJ. Participation levels of physical activity programs for community-dwelling older adults: a systematic review. BMC Public Health. (2014) 14:1301. doi: 10.1186/1471-2458-14-1301, PMID: 25523712 PMC4301079

[26] Tischer U Hartmann-Tews I Combrink C. Sport participation of the elderly—the role of gender, age, and social class. Eur Rev Aging Phys Act. (2011) 8:83–91. doi: 10.1007/s11556-011-0087-8

[27] Martínez del Castillo J González MD Jiménez-Beatty JE Graupera JL Martín M Campos A. Los hábitos de actividad física de las mujeres mayores en España. Rev Int Cienc Deporte. (2009) 5:81–93. doi: 10.5232/ricyde2009.01407

[28] Martins LCG Lopes MVO Diniz CM Guedes NG. The factors related to a sedentary lifestyle: a meta-analysis review. J Adv Nurs. (2021) 77:1188–205. doi: 10.1111/jan.14669 33368524

[29] Trapé A Marques RFR Lizzi EAS Yoshimura FE Franco LJ Zago AS. Association between demographic and socioeconomic conditions with exercise practice and physical fitness in community projects participants aged 50 years or more in Ribeirão Preto, São Paulo. Rev Bras Epidemiol. (2017) 20:355–67. doi: 10.1590/1980-5497201700020015, PMID: 28832857

[30] Ballesteros MS Freidin B Wilner A Fernández Rendina L. Interseccionalidad en las desigualdades sociales para la realización de actividad física en Argentina. Rev Cienc de la Salud. (2020) 18:134–51. doi: 10.12804/revistas.urosario.edu.co/revsalud/a.8777

[31] Delpino FM de Lima APM da Silva BGC Nunes BP Caputo EL Bielemann RM. Physical activity and multimorbidity among community-dwelling older adults: a systematic review with Meta-analysis. Am J Health Promot. (2022) 36:1371–85. doi: 10.1177/08901171221104458, PMID: 35621359

[32] Bullard T Ji M An R Trinh L Mackenzie M Mullen SP. A systematic review and meta-analysis of adherence to physical activity interventions among three chronic conditions: Cancer, cardiovascular disease, and diabetes. BMC Public Health. (2019) 19:1–11. doi: 10.1186/s12889-019-6877-z, PMID: 31126260 PMC6534868

[33] Serrano-Sánchez JA Bello-Luján LM Auyanet-Batista JM Fernández-Rodríguez MJ González-Henríquez JJ. Lack of exercise of "moderate to vigorous" intensity in people with low levels of physical activity is a major discriminant for sociodemographic factors and morbidity. PLoS One. (2014) 9:e115321. doi: 10.1371/journal.pone.0115321, PMID: 25522144 PMC4270757

[34] Ribeiro AQ Salgado SML Gomes IS Fogal AS Martinho KO Almeida LFF. Prevalence and factors associated with physical inactivity among the elderly: a population-based study. RBGG. (2016) 19:483–93. doi: 10.1590/1809-98232016019.150047

[35] Val Jiménez CL López-Torres Hidalgo J García Atienza EM Navarro Ruiz MS Hernández Cerón I Moreno de la Rosa L. Situación funcional, autopercepción de salud y nivel de actividad física en pacientes con artrosis. Aten Primaria. (2017) 49:224–32. doi: 10.1016/j.aprim.2016.06.002, PMID: 27469248 PMC6875987

[36] Penton H Dayson C Hulme C Young T. A qualitative investigation of older adults’ conceptualization of quality of life and a think-aloud content validation of the EQ-5D-5L, SF-12v2, Warwick Edinburgh mental well-being scale, and Office of National Statistics-4. Value Health. (2022) 25:2017–27. doi: 10.1016/j.jval.2022.04.1735, PMID: 35760713

[37] Franco-García JM Denche-Zamorano Á Pereira-Payo D Rodríguez-Redondo Y Carlos-Vivas J Castillo-Paredes A. Association between GHQ-12, Duke-UNC-11, physical activity, and self-perceived health in Spanish adults with cancerous Tumours: a cross-sectional study. Healthcare. (2023) 11:192. doi: 10.3390/healthcare11020192, PMID: 36673560 PMC9858809

[38] Essery R Geraghty AWA Kirby S Yardley L. Predictors of adherence to home-based physical therapies: a systematic review. Disabil Rehabil. (2017) 39:519–34. doi: 10.3109/09638288.2016.1153160, PMID: 27097761

[39] Mas-Pons R Barona-Vilar C Ninyoles G García AM. Salud en todas las políticas en la Comunitat Valenciana: pasos hacia la evaluación del impacto en salud. Gac Sanit. (2019) 33:593–7. doi: 10.1016/j.gaceta.2018.09.002, PMID: 30553532

[40] Husebø AML Dyrstad SM Søreide JA Bru E. Predicting exercise adherence in cancer patients and survivors: a systematic review and meta-analysis of motivational and behavioural factors. J Clin Nurs. (2013) 22:4–21. doi: 10.1111/j.1365-2702.2012.04322.x, PMID: 23163239

[41] Burgess E Hassmén P Pumpa KL. Determinants of adherence to lifestyle intervention in adults with obesity: a systematic review. Clin Obes. (2017) 7:123–35. doi: 10.1111/cob.12183, PMID: 28296261

[42] Bermejo JP Almagro BJ Rebollo JA. Factores motivacionales relacionados con la intención de seguir practicando ejercicio físico en mujeres adultas. Retos. (2018) 34:117–22. doi: 10.47197/retos.v0i34.50748

